# Pregnancy outcomes following fluoroscopy-guided tubal recanalization: a comparison of spontaneous conception and intrauterine insemination—a retrospective cohort study

**DOI:** 10.1038/s41598-025-17762-z

**Published:** 2025-09-01

**Authors:** Ceyda Karadag, Burak Karadag, Emine Yildiz Kugu Kesen, Cemil Gürses

**Affiliations:** 1 Department of Obstetrics and Gynecology, Saglık Bilimleri University Antalya City Hospital, Antalya, Turkey; 2 Department of Obstetrics and Gynecology, Saglık Bilimleri University Antalya Training and Research Hospital, Antalya, Turkey; 3 Department of Radiology, Saglık Bilimleri University Antalya Training and Research Hospital, Antalya, Turkey

**Keywords:** Intrauterine insemination (IUI), Spontaneous conception, Tubal factor infertility, Tubal recanalization, Infertility, Urogenital reproductive disorders

## Abstract

To evaluate and compare pregnancy outcomes following successful fluoroscopy-guided tubal recanalization (FGTR), focusing on spontaneous conception versus intrauterine insemination (IUI). This retrospective cohort study included 139 women aged 21–40 years who underwent FGTR for tubal occlusion between January 2021 and May 2024. After exclusions, 80 women attempted natural conception, and 59 underwent IUI with ovarian stimulation. Groups were compared in terms of clinical pregnancy rates, time to conception, and post-procedure tubal patency. Clinical pregnancy rates were similar between spontaneous conception (51.2%) and IUI (57.6%) groups (p = 0.976). Mean time to conception did not differ significantly (6.4 ± 2.8 vs. 5.9 ± 2 months, p = 0.360). No pregnancies occurred in women with bilateral distal obstruction, whereas proximal occlusions (unilateral or bilateral) were associated with higher pregnancy rates. Six-month follow up HSG demonstrated that bilateral tubal patency correlated with greater conception likelihood. In women with tubal factor infertility who achieve patency after FGTR, spontaneous conception and IUI yield comparable pregnancy outcomes and similar time to conception. Expectant management may be a cost-effective first-line approach, reserving IUI or IVF for cases without conception within a reasonable timeframe. The site of tubal obstruction and patency status should guide individualized post-FGTR fertility planning.

## Introduction

Infertility affects approximately 10–15% of couples worldwide, with tubal factor infertility accounting for nearly 30–40% of cases^1^. In women, the most frequent causes include ovulatory disorders and problems related to the fallopian tubes^2^. Tubal factor infertility is a significant contributor, estimated to be responsible for 11% to 67% of infertility diagnoses^2^. This type of infertility can arise from blockages within the fallopian tubes, structural abnormalities resulting from inflammation or infection, or pelvic adhesions that hinder the normal function of the tubes.

Fallopian tube blockages can occur near the uterus (proximally). These proximal blockages often consist of accumulated debris forming a plug^3^. Blockages further down the tube (distally) are typically the result of prior infections, potentially leading to hydrosalpinx, characterized by a widened fallopian tube and the absence of dye spillage during a hysterosalpingography (HSG)^4^. Tubal occlusion can result from infections, pelvic inflammatory disease, endometriosis, or prior surgical interventions^5^. Fluoroscopy-guided tubal recanalization (FGTR), a minimally invasive procedure, has emerged as an effective treatment for restoring tubal patency and improving the chances of natural conception^6^.

The optimal management strategy after FGTR, whether to pursue natural conception or use fertility interventions such as intrauterine insemination (IUI) remains a topic of debate. Although both methods are widely used, there is limited evidence directly comparing their effectiveness in women who have undergone FGTR. Understanding the relative success rates and time to conception between these two approaches is essential for optimizing fertility treatment strategies.

This study aims to evaluate pregnancy outcomes following FGTR, comparing spontaneous conception rates and pregnancy outcomes achieved through IUI.

## Materials and methods

### Patient population and study design

Patients were identified from a prospectively maintained database of women undergoing FGTR at our institution. The study was approved by the Institutional Review Board. All patients provided informed consent for the procedure and data analysis.

### Inclusion and exclusion criteria

The inclusion criteria for this study were as follows: women aged between 21 and 40 years; a diagnosis of unilateral or bilateral tubal occlusion confirmed by (HSG) or laparoscopy; successful FGTR of at least one fallopian tube, defined as tubal patency demonstrated by post-procedure HSG or laparoscopic chromopertubation; normal ovulatory function, as evidenced by regular menstrual cycles and mid-luteal serum progesterone levels; and a partner with normal semen parameters according to the World Health Organization (WHO) criteria^7^. Not all patients in our study underwent diagnostic laparoscopy. However, for the subgroup of women who did undergo laparoscopy due to clinical suspicion of pelvic pathology, any detected adhesions or structural abnormalities were surgically treated during the same session. Importantly, only those patients with persistent tubal occlusion confirmed by laparoscopic chromopertubation after adhesiolysis or other surgical corrections were included in the study. If chromopertubation demonstrated tubal patency following the surgical intervention, such patients were excluded from our analysis, as they no longer fulfilled the inclusion criterion of tubal occlusion. Thus, only cases with confirmed and persistent tubal blockage post-adhesiolysis were considered eligible.

**Fig. 1:**
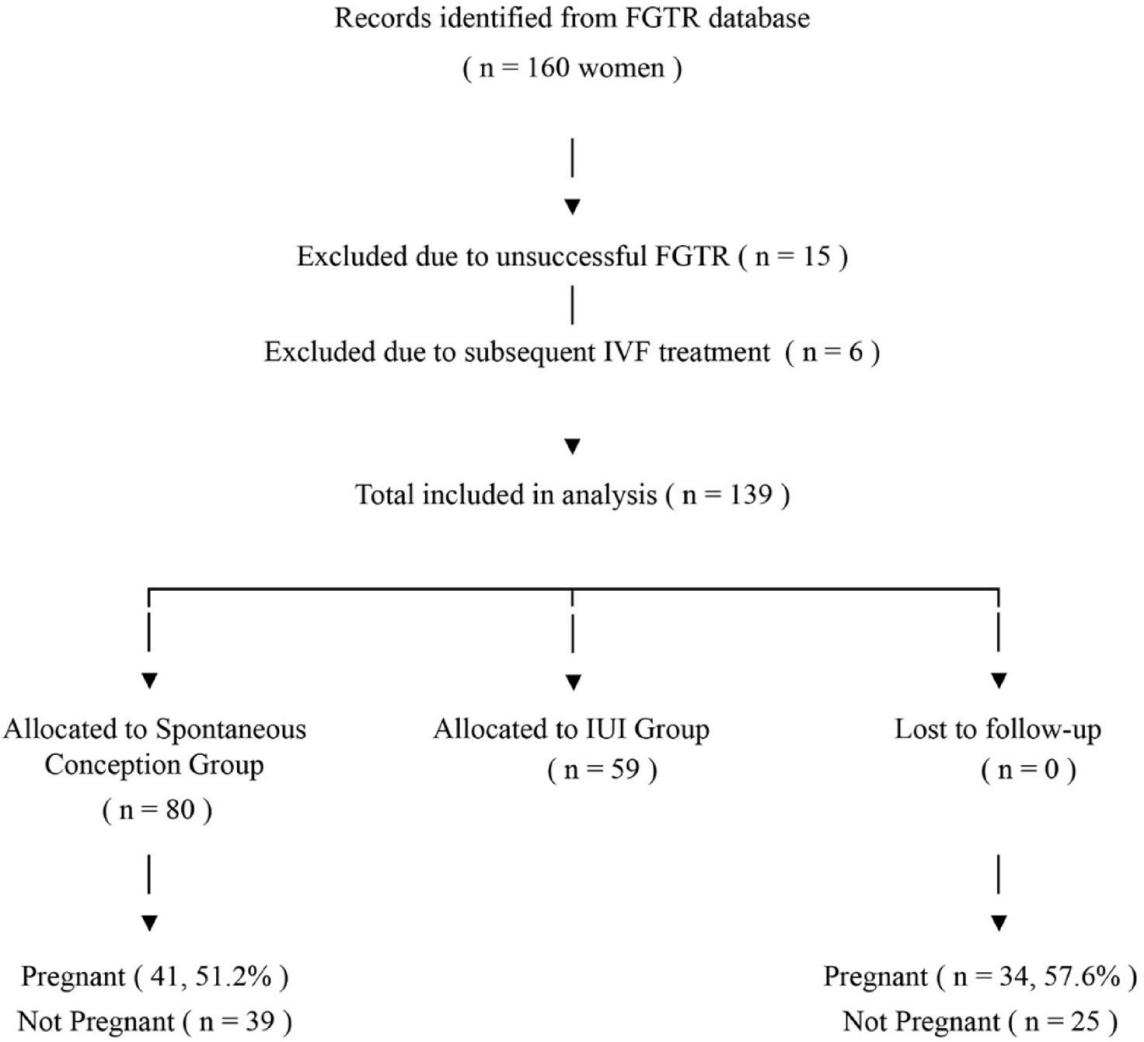
Flowchart of patient selection, inclusion, exclusion, and allocation to treatment groups.

Exclusion criteria included bilateral tubal occlusion not amenable to recanalization as determined by pre-operative imaging; the presence of significant pelvic adhesions, endometriosis, or other pelvic comorbidities identified during laparoscopy; a history of conception via in vitro fertilization (IVF); and inability to complete follow up for at least 12 months after recanalization. Patient selection and flow through the study process are illustrated in Fig. 1.

### Fluoroscopy-guided tubal recanalization (FGTR) equipment and technique

The procedure was performed using a digital subtraction angiography system (Canon INFX-8000 V) available in the Department of Radiology. All FGTR procedures in this study were performed by a single experienced interventional radiologist using a standardized protocol. Tubal recanalization was performed using a 5F angled vertebral catheter (0.046″), a 0.035″ hydrophilic guidewire, and, if necessary, a 3F micro-catheter (0.027″) with a 0.018″ micro-guidewire.

Following the application of an antiseptic disinfectant to the skin/genital area, a speculum is inserted. Once the cervical external os is visualized, a catheter is placed into the uterine cavity under aseptic conditions. The previously detected tubal occlusion on HSG is reassessed by administering contrast medium under fluoroscopic guidance.

**Fig. 2:**
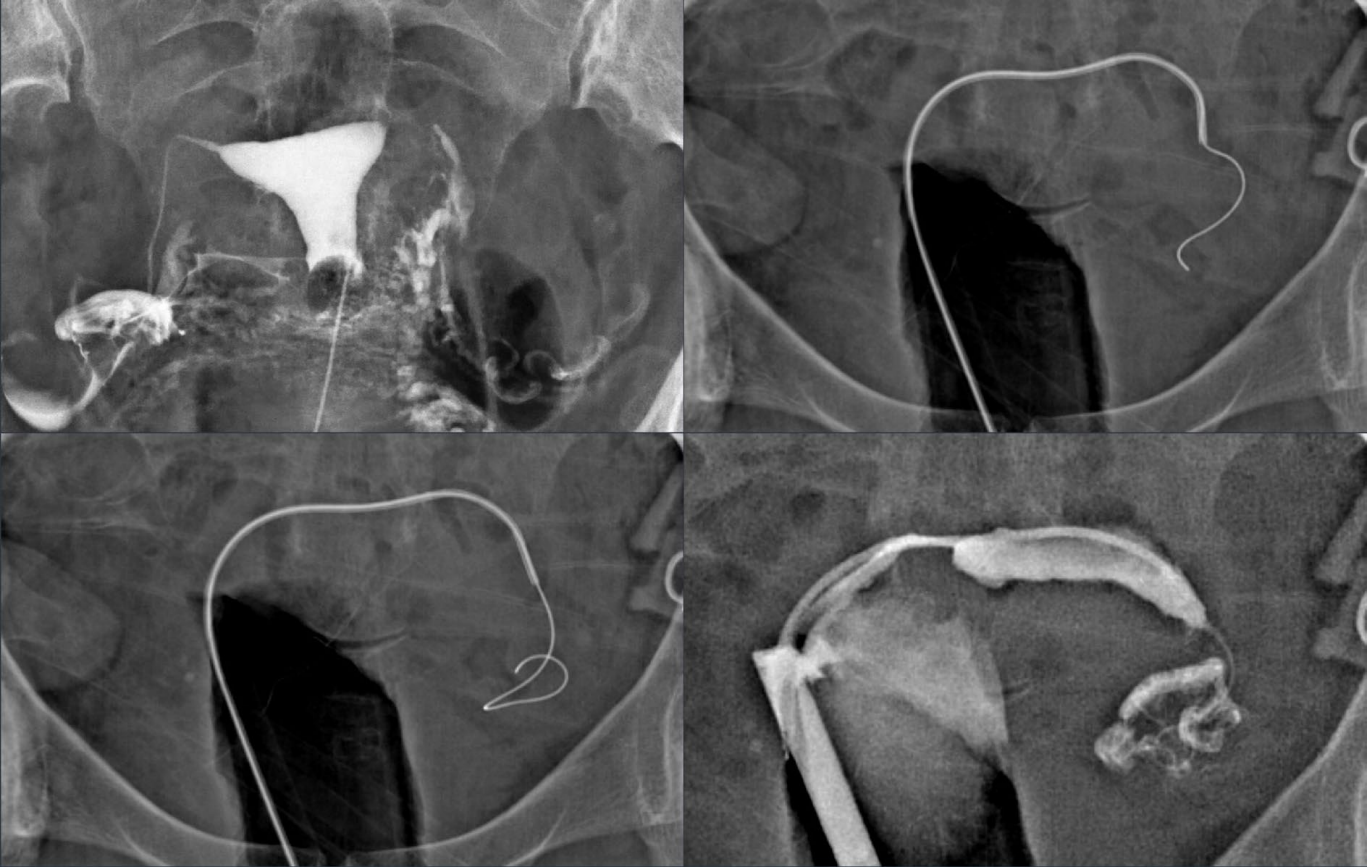
Fluoroscopic view demonstrating recanalization technique for left proximal tubal occlusion.

A 5F, 0.046 angled-tip catheter is then advanced to the tubal ostium, and a 0.035 hydrophilic guidewire is introduced. The guidewire is carefully manipulated under real-time fluoroscopy until it advances beyond the proximal two-thirds of the fallopian tube. Once positioned, the guidewire is removed, and contrast medium is injected through the catheter to evaluate peritoneal contrast spillage, confirming tubal patency. Figure 2 illustrates the recanalization technique used for proximal tubal occlusion.

**Fig. 3:**
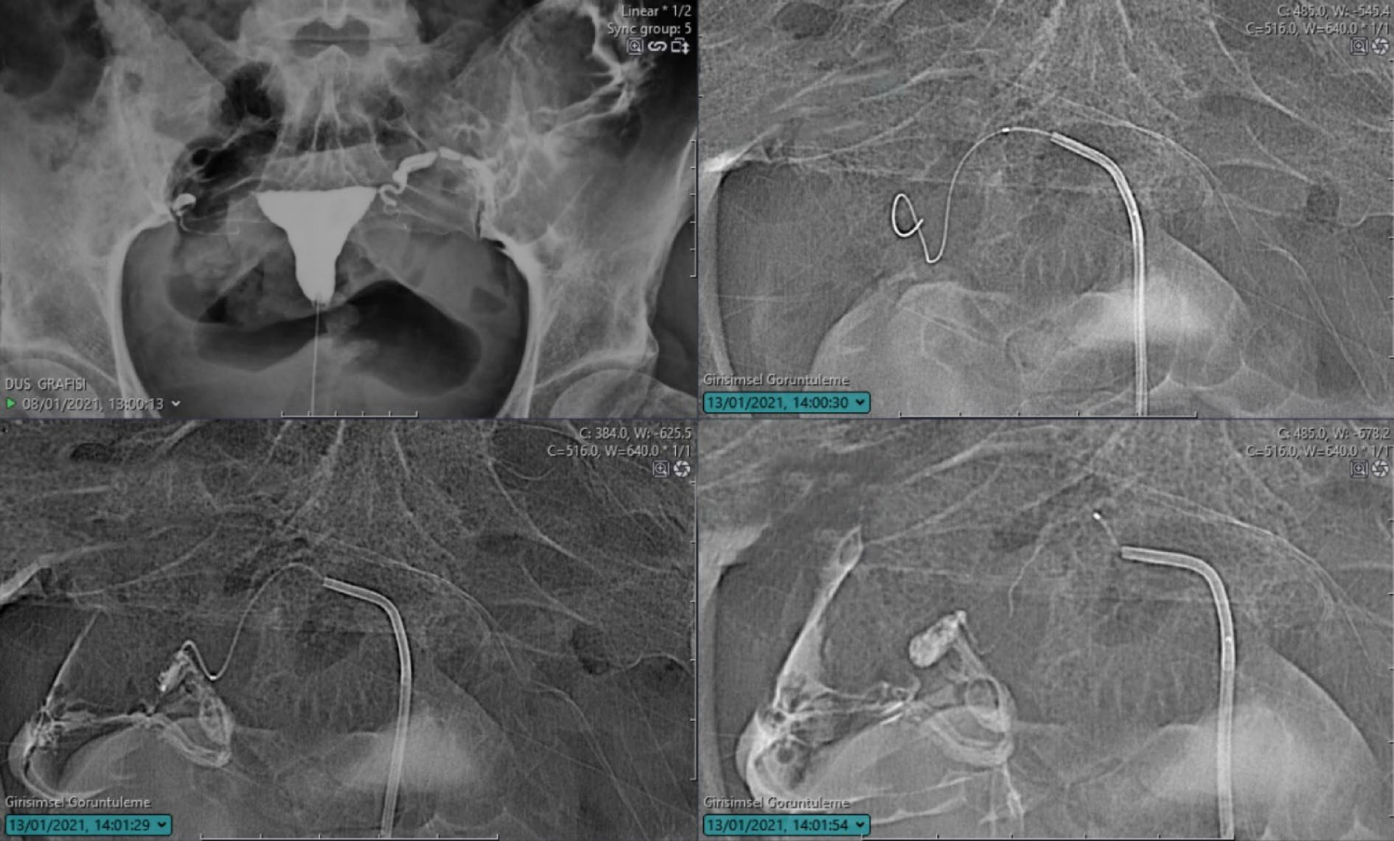
Fluoroscopic view demonstrating recanalization technique for right distal tubal occlusion.

If the 0.035 hydrophilic guidewire is unable to traverse the occlusion, a micro catheter and micro-guidewire system is introduced through the 5F catheter positioned at the tubal ostium. Under fluoroscopic guidance, the micro-guidewire is carefully advanced through the tubal lumen, followed by the micro catheter. Once the micro catheter reaches the desired location, the micro-guidewire is withdrawn, and contrast medium is injected to assess peritoneal passage. Figure 3 demonstrates the recanalization technique for distal tubal occlusion.

Following the procedure, the patient is prescribed doxycycline and is observed briefly before discharge. Additionally, if pregnancy does not occur by the 6th month post-cannulation, an HSG is performed to evaluate tubal patency.

*Treatment Groups*: Following successful FGTR, patients were counseled regarding options for conception, including expectant management (spontaneous conception group) and intrauterine insemination (IUI group). The choice of treatment was based on patient preference and physician recommendation, considering factors such as age, duration of infertility, and semen parameters. The spontaneous conception group (*n* = 80) attempted to conceive naturally, while the IUI group (*n* = 59) underwent IUI cycles with ovarian stimulation using clomiphene citrate or letrozole.

*Outcome Measures*: The primary outcome measure was the positive clinical pregnancy rate, defined as the presence of a gestational sac on ultrasound. Secondary outcome measures included time to pregnancy (in months) and the association between the type of tubal occlusion (e.g., proximal vs. distal) and pregnancy outcomes.

*Statistical Analysis*: Statistical analysis was performed using SPSS version 26.0 (IBM Corp., Armonk, NY). Continuous variables were expressed as mean ± standard deviation and compared using Student’s *t*-test. Categorical variables were expressed as frequencies and percentages and compared using the chi-square test or Fisher’s exact test, as appropriate. Pregnancy rates over a 12-month period were analyzed using Kaplan–Meier survival analysis, and differences between groups were assessed with the log-rank test. A *p*-value of < 0.05 was considered statistically significant.

## Results

### Patient characteristics

This retrospective cohort study evaluated pregnancy outcomes in 139 women aged 21–40 years who underwent successful FGTR for tubal occlusion between January 2021 and May 2024. After excluding 15 patients with unsuccessful recanalization and 6 patients who subsequently underwent IVF, 80 patients were included in the spontaneous conception group and 59 in the IUI group. The mean operative time was 12.1 ± 2.31 min for unilateral recanalization and 24.7 ± 4.4 min for bilateral recanalization. Baseline characteristics of the two groups are summarized in Table 1. There were no statistically significant differences between the two groups with respect to age (32.2 ± 4.5 vs. 31.6 ± 4.1 years, *p* = 0.397), parity [1 (0–3) vs 1 (0–2), p = 0.085], type of infertility (primary: 26 (32.5%) vs. 24 (40.7%), *p* = 0.321), duration of infertility (16.1 ± 3.3 vs. 16.8 ± 3.6 months, *p* = 0.818), site of obstruction (Bilateral: 44 (55%) vs 35 (59.3%), *p* = 0.801) or type of obstruction (Proximal: 39 (48.8%) vs 35 (59.3%), *p* = 0.217).

**Table 1:** Baseline characteristics.

		Spontaneous (*n* = 80)	IUI (*n* = 59)	*p*
Age (years)		32.2 ± 4.5	31.6 ± 4.1	0.397
Parity	0	32 (40%)	28 (47.5%)	0.210
	1	26 (32.5%)	23 (39%)	
	2	20 (25%)	8 (13.6%)	
	3	2 (2.5%)	-	
Type of infertility	Primary	26 (32.5%)	24 (40.7%)	0.321
	Secondary	54 (67.5%)	35 (59.3%)	
Duration of infertility	12 to 24 months	78 (97.5%)	57 (96.6%)	0.756
	24 to 36 months	2 (2.5%)	2 (3.4%)	
Type of obstruction	Unilateral Proximal	24 (30%)	17 (28.8%)	0.505
	Unilateral Distal	12 (15%)	7 (11.9%)	
	Bilateral proximal	32 (40%)	30 (50.8%)	
	Bilateral Distal	12 (15%)	7 (11.9%)	
Positive pregnancy	41 (51.2%)	34 (57.6%)	0.976	
Time to pregnancy (months)	6.4 ± 2.8	5.9 ± 2	0.360	
6 month tubal patency	Bilateral patency	39 (48.8%)	27 (45.8%)	0.820
	Unilateral Patency	12 (15%)	8 (13.6%)	
	No patency	3 (3.8%)	1 (1.7%)	

IUI = intrauterine insemination; HSG = hysterosalpingography.

Table 2 highlights the association between the type of tubal obstruction and 6-month post-recanalization patency. It shows that women with bilateral proximal obstruction had a significantly higher rate of bilateral patency (50%) compared to those with unilateral patency (30%) and no patency (0%) (p = 0.001).

**Table 2:** Association between type of tubal obstruction and 6 month post-recanalization.

Obstruction type	6-month patency on HSG							*p*^2^
	Bilateral Patency			Unilateral Patency*		No Patency*		
	S (*n*= 39)	IUI (*n*= 27)	p^1^	S (*n* = 12)	IUI (*n* = 8)	S (*n* = 3)	IUI (*n* = 1)	
Bilateral proximal	18(46.2%)	15(55.6%)	0.238	4(33.3%)	2(25%)			0.001
Bilateral distal	3(7.7%)	9 (33.3%)		6(50%)	4(50%)	3(100%)	1(100%)	
Unilateral proximal	9(23.1%)	3(11.1%)		1(8.3%)	-			
Unilateral distal	9(23.1%)	–		1(8.3%)	2(25%)			

HSG = hysterosalpingography, S = Spontaneous conception group, IUI = Intrauterine insemination group * Statistical comparison was not performed due to insufficient sample size in the respective groups.

p^1^: p-value for the comparison between spontaneous conception and IUI groups within the bilateral patency category for a given obstruction type.

p^2^: Overall p-value for the association between obstruction type and patency status in the entire cohort (S + IUI combined).

### Pregnancy outcomes

**Fig. 4:**
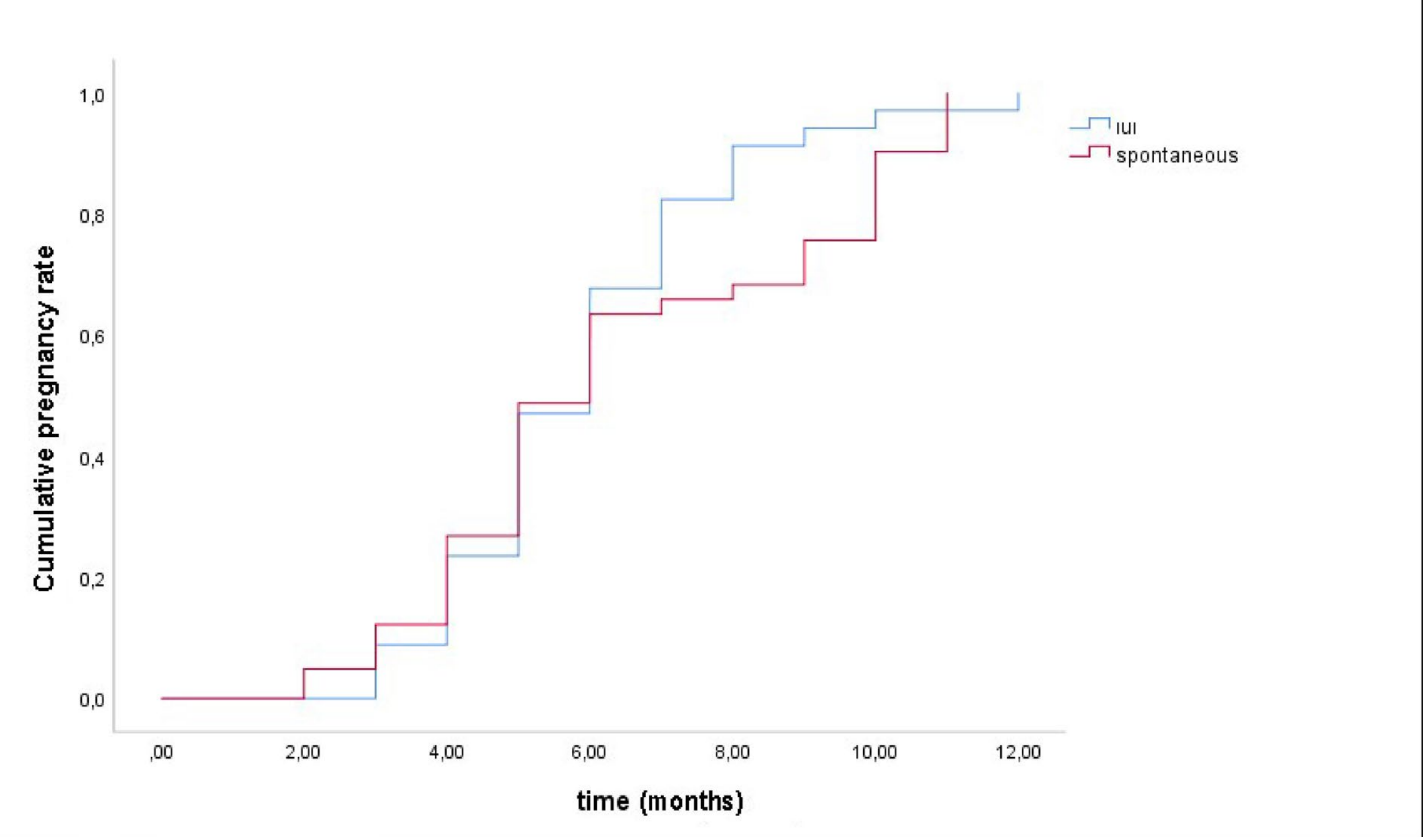
Comparison of pregnancy rates within the first 12 months following tubal recanalization.

The positive pregnancy rate was 51.2% (41/80) in the spontaneous conception group and 57.6% (34/59) in the IUI group (*p* = 0.976). The mean time to pregnancy was 6.4 ± 2.8 months in the spontaneous conception group and 5.9 ± 2 months in the IUI group (*p* = 0.360). Analysis of pregnancy outcomes based on tubal obstruction type revealed significant findings (Table 3). No pregnancies occurred among women with bilateral distal obstruction (*p* = 0.001). In contrast, patients with unilateral proximal obstruction (46.7%) and bilateral proximal obstruction (45.3%) demonstrated higher pregnancy rates. Women with distal occlusions, especially bilateral, were significantly less likely to conceive. Figure 4 shows pregnancy rates within the first 12 months following tubal recanalization.

**Table 3:** Comparison of obstruction types in pregnant and non-pregnant patients.

Obstruction type	Pregnant (n = 75)			No pregnancy (n = 64)			*p*^3^
	S (*n*= 41)	IUI (*n* = 34)	p^1^	S (*n* = 39)	IUI (*n* = 25)	p^2^	
Bilateral proximal	16(39%)	18(52.9%)	0.164	16(41%)	12(48%)	0.067	0.001
Bilateral distal	-–	–		12(30.8%)	5(20%)		
Unilateral proximal	23(56.1%)	12(35.3%)		1(2.6%)	5(20%)		
Unilateral distal	2(4.9%)	4(11.8%)		10(25.6%)	3(12%)		

S = Spontaneous conception group, IUI = Intrauterine insemination group.

p^1^: p-value for the comparison between S and IUI groups within the pregnant category for each obstruction type.

p^2^: p-value for the comparison between S and IUI groups within the no pregnancy category for each obstruction type.

p^3^: Overall p-value for the association between obstruction type and pregnancy outcome in the entire cohort (S + IUI combined).

No major complications related to the recanalization procedure were reported.

## Discussion

This study examined pregnancy outcomes in women who underwent FGTR for tubal occlusion, comparing spontaneous conception with intrauterine insemination (IUI). Both strategies yielded similar pregnancy rates (51.2% vs. 57.6%) and time to conception, indicating that in appropriately selected patients, natural conception after FGTR can be as effective as IUI. This is particularly relevant when tubal pathology is the only infertility factor, as recanalization directly addresses the primary barrier to conception. In such cases, the absence of other infertility causes may explain the comparable outcomes between spontaneous and assisted conception. These findings are consistent with previous literature reporting post-recanalization spontaneous pregnancy rates of 30–60% without further interventions^8–10^.

Pregnancy outcomes in our cohort varied according to both the laterality and site of obstruction. Proximal occlusions—often functional in origin and related to mucus plugs or debris—showed favorable outcomes after FGTR, in line with earlier reports^11,12^. In our data, unilateral and bilateral proximal obstructions achieved pregnancy rates of 46.7% and 45.3%, respectively. In contrast, distal disease—especially when bilateral—was associated with markedly lower pregnancy rates, and no pregnancies occurred in women with bilateral distal occlusion. Unilateral distal obstruction also reduced fertility potential, consistent with findings by Lin et al.^13^. These differences likely reflect the irreversible structural damage, fibrosis, and ciliary loss common in distal pathology^4,11,13^.

Although IVF is widely recommended for distal disease, catheter-based recanalization is not an absolute contraindication. Several studies have demonstrated feasibility and occasional success in distal interventions. Sueoka et al. achieved pregnancies after falloposcopic tuboplasty in bilateral cases including distal involvement^14^, and Li et al. reported outcomes following hysteroscopic tubal catheterization and hydrotubation^15^. Allahbadia and Merchant emphasized that, while technically demanding and prognostically less favorable, distal recanalization can be justified in carefully selected patients who understand the limitations and prefer a less invasive option before proceeding to IVF^4^. In our center, such patients were thoroughly counseled, informed consent was obtained, and alternative options discussed. Including these cases allowed us to present real-world outcome data for a subgroup often excluded from studies, thereby helping to inform future patient counseling.

Importantly, women with unilateral tubal occlusion comprised a significant portion of our cohort. This is noteworthy because existing literature indicates that pregnancy rates remain relatively high in such patients, even without intervention on the occluded side. In cases of unilateral blockage, the contralateral patent tube can often compensate functionally, allowing for natural conception, especially in women with normal ovulatory cycles and favorable semen parameters^13^. Nevertheless, several studies have indicated that recanalization of the occluded tube may further improve reproductive outcomes even in this context. Schill et al. demonstrated better outcomes in unilateral proximal obstruction compared to bilateral disease, while Hayashi et al. found high pregnancy rates when the contralateral tube was patent^11,12^. Our results support these observations and highlight that both laterality and anatomical location should guide post-FGTR management strategies. Patients with unilateral proximal occlusion may be ideal candidates for expectant management, whereas those with distal involvement—regardless of laterality—require closer follow up and potentially earlier transition to assisted reproductive technologies.

Follow up imaging proved valuable in prognostication. Six-month HSG demonstrated that bilateral patency correlated with higher pregnancy rates, while recurrent or persistent occlusion predicted poorer outcomes. This supports the recommendation by Schankath et al. for repeat HSG within 3–6 months to guide timely IVF referral^16^.

From a clinical perspective, FGTR offers several advantages: it is minimally invasive, performed under fluoroscopic guidance without the need for general anesthesia, and has a short recovery period. For proximal obstruction, it can restore fertility without resorting to more invasive surgery or expensive ART. However, FGTR has limitations. Technical difficulty increases with complex or distal lesions, and the procedure requires specialized equipment and operator expertise, limiting availability. Radiation exposure, while generally low with modern techniques, remains a consideration for reproductive-aged women, particularly if multiple imaging procedures are needed.

Alternative treatments include laparoscopic tuboplasty or tubal re-anastomosis, which may be indicated in selected distal disease or post-sterilization reversal cases. These surgical options, however, require general anesthesia, involve longer recovery times, and carry higher procedural risks. IVF remains the most effective approach for advanced distal disease or in cases with poor tubal mucosal integrity, but it is costlier, invasive, and emotionally demanding.

The strengths of our study include a relatively large, well-defined cohort and the fact that all FGTR procedures were performed by a single experienced interventional radiologist, minimizing operator variability. Strict inclusion criteria ensured a homogeneous study population. Limitations include the retrospective design, potential selection bias due to non-randomized treatment allocation, and relatively short follow up limited to pregnancy rather than live birth rates.

In summary, FGTR is an effective, minimally invasive option for women with tubal factor infertility, particularly for proximal disease. In carefully selected patients without other infertility factors, spontaneous conception following FGTR can be considered a first-line approach, with IUI or IVF reserved for those who do not conceive within a reasonable timeframe. Anatomical site and laterality of obstruction, as well as follow up patency assessment, should guide individualized treatment planning to optimize reproductive outcomes.

## Conclusion

FGTR is an effective treatment for women with tubal occlusion. Both spontaneous conception and IUI offer comparable pregnancy outcomes, with no significant differences in pregnancy rates or time to conception. This suggests that spontaneous conception may be a viable first-line option, with IUI reserved for patients who do not conceive within a certain period. Further studies are needed to identify predictors of success in these groups. Specifically, research should focus on identifying which patient characteristics (e.g., age, duration of infertility, specific tubal pathology) are most predictive of success with spontaneous conception versus IUI.

## Data Availability

The datasets used and/or analyzed during the current study are available from the corresponding author on reasonable request.

## Abbreviations

BMI: Body Mass Index
E2: Estradiol
FGTR: Fluoroscopy-Guided Tubal Recanalization
FSH: Follicle-Stimulating Hormone
hCG: Human Chorionic Gonadotropin
HSG: Hysterosalpingography
IUI: Intrauterine Insemination
IVF: In Vitro Fertilization
LH: Luteinizing Hormone
PCOS: Polycystic Ovary Syndrome
PID: Pelvic Inflammatory Disease
PRL: Prolactin
TSH: Thyroid-Stimulating Hormone
TVUSG: Transvaginal Ultrasonography

## Declarations

### What does this study add to the clinical work

This study highlights the clinical value of tubal fluoroscopy-guided tubal recanalization (FGTR) as a minimally invasive, fertility-enhancing intervention in patients with proximal tubal occlusion. It demonstrates that successful recanalization not only restores tubal patency but also significantly improves spontaneous pregnancy rates and outcomes following intrauterine insemination (IUI). These findings support incorporating FGTR into routine fertility assessments and treatment plans, potentially reducing the need for more invasive or costly assisted reproductive techniques such as IVF.

## References

[1] Zegers-Hochschild, F. et al. The international glossary on infertility and fertility care. *Fertil Steril.* **108**(3), 393–406 (2017). 28760517 10.1016/j.fertnstert.2017.06.005

[2] Carson, S. A. & Kallen, A. N. Diagnosis and management of infertility: A review. *JAMA* **326**(1), 65–76 (2021). 34228062 10.1001/jama.2021.4788 PMC9302705

[3] Das, S. Proximal tubal disease: the place for tubal cannulation. *Reprod Med* **15**, 383–388 (2007). 10.1016/s1472-6483(10)60362-8 17908398

[4] Allahbadia, G. N. & Merchant, R. Fallopian tube recanalization: lessons learnt and future challenges. *Women’s Health* **6**, 531–549 (2010). 20597618 10.2217/whe.10.34

[5] Practice Committee of the American Society for Reproductive Medicine. Fertility evaluation of infertile women: A committee opinion. *Fertil Steril.* **116**(5), 1255–1265 (2021). 34607703 10.1016/j.fertnstert.2021.08.038

[6] Tanaka, Y. & Tajima, H. Falloposcopic tuboplasty as an option for tubal infertility: an alternative to in vitro fertilization. *Fertil Steril.* **95**(1), 441–443 (2011). 20797702 10.1016/j.fertnstert.2010.07.1065

[7] World Health Organization. *WHO laboratory manual for the examination and processing of human semen* 6th edn. (World Health Organization, 2021).

[8] Thurmond, A. S. Fallopian tube catheterization. *Semin Intervent Radiol.* **30**(4), 381–387 (2013). 24436565 10.1055/s-0033-1359732 PMC3835434

[9] Mody, P., Salazar, G. & Kohi, M. P. Recanalization of proximal fallopian tube obstruction in the treatment of infertility. *Semin Intervent Radiol.* **40**(4), 379–383 (2023). 37575349 10.1055/s-0043-1771042 PMC10415059

[10] Wang, J. W. et al. Conception rates after fluoroscopy-guided fallopian tubal cannulation: an alternative to in vitro fertilization for patients with tubal occlusion. *Ther Adv Reprod Health.* **8**(14), 2633494120954248 (2020). 10.1177/2633494120954248 PMC7549323 33103116

[11] Schill, T. et al. Transcervical falloscopic dilatation of proximal tubal occlusion. Is there an indication?. *Hum Reprod.* **14**(1), 137–144 (1999). 10573030 10.1093/humrep/14.suppl_1.137

[12] Hayashi, M., Hoshimoto, K. & Ohkura, T. Successful conception following Fallopian tube recanalization in infertile patients with a unilateral proximally occluded tube and a contralateral patent tube. *Hum Reprod.* **18**(1), 96–99 (2003). 12525447 10.1093/humrep/deg006

[13] Lin, M. H., Hwu, Y. M., Lin, S. Y. & Lee, R. K. Treatment of infertile women with unilateral tubal occlusion by intrauterine insemination and ovarian stimulation. *Taiwan J Obstet Gynecol.* **52**(3), 360–364 (2013). 24075374 10.1016/j.tjog.2012.01.037

[14] Sueoka, K. et al. Falloposcopic tuboplasty for bilateral tubal occlusion. A novel infertility treatment as an alternative for in-vitro fertilization?. *Hum Reprod.* **13**(1), 71–74 (1998). 9512231 10.1093/humrep/13.1.71

[15] Li, S. C., Liu, M. N., Hu, X. Z. & Lu, Z. L. Hysteroscopic tubal catheterization and hydrotubation for treatment of infertile women with tubal obstruction. *Chin Med J (Engl).* **107**(10), 790–793 (1994). 7835109

[16] Schankath, A. C., Fasching, N., Urech-Ruh, C., Hohl, M. K. & Kubik-Huch, R. A. Hysterosalpingography in the workup of female infertility: Indications, technique and diagnostic findings. *Insights Imaging.* **3**(5), 475–483 (2012). 22802083 10.1007/s13244-012-0183-y PMC3443271

